# Case Report: Novel compound heterozygous variants in *CHRNA1* gene leading to lethal multiple pterygium syndrome: A case report

**DOI:** 10.3389/fgene.2022.964098

**Published:** 2022-08-26

**Authors:** Jianlong Zhuang, Junyu Wang, Qi Luo, Shuhong Zeng, Yu’e Chen, Yuying Jiang, Xinying Chen, Yuanbai Wang, Yingjun Xie, Gaoxiong Wang, Chunnuan Chen

**Affiliations:** ^1^ Prenatal Diagnosis Center, Quanzhou Women’s and Children’s Hospital, Quanzhou, China; ^2^ Department of Public Health for Women and Children, Quanzhou Women’s and Children’s Hospital, Quanzhou, China; ^3^ Ultrasonography, Quanzhou Women’s and Children’s Hospital, Quanzhou, China; ^4^ Department of Obstetrics and Gynecology, Guangdong Provincial Key Laboratory of Major Obstetric Diseases, The Third Affiliated Hospital of Guangzhou Medical University, Guangzhou, China; ^5^ Key Laboratory of Reproduction and Genetics of Guangdong Higher Education Institutes, The Third Affiliated Hospital of Guangzhou Medical University, Guangzhou, China; ^6^ Quanzhou Women’s and Children’s Hospital, Quanzhou, China; ^7^ Department of Neurology, The Second Affiliated Hospital of Fujian Medical University, Quanzhou, China

**Keywords:** whole-exome sequencing, chromosomal microarray analysis, *CHRNA1*, lethal multiple pterygium syndrome, stillbirth

## Abstract

**Background:** Lethal multiple pterygium syndrome (LMPS) is a rare autosomal recessive inherited disorder typically characterized by intrauterine growth retardation, multiple pterygia, and flexion contractures.

**Case presentation:** We herein report a Chinese case with a history of three adverse pregnancies demonstrating the same ultrasonic phenotypes, including increased nuchal translucency, edema, fetal neck cystoma, reduced movement, joint contractures, and other congenital features. Whole-exome sequencing (WES) revealed novel compound heterozygous variants in the *CHRNA1* gene NM_000079.4: c.[1128delG (p.Pro377Leu*fs*Ter10)]; [505T>C (p.Trp169Arg)] in the recruited individual, and subsequent familial segregation showed that both parents transmitted their respective mutation.

**Conclusion:** For the first time, we identified an association between the *CHRNA1* gene and the recurrent lethal multiple pterygium syndrome (LMPS) in a Chinese family. This finding may also enrich the mutation spectrum of the *CHRNA1* gene and promote the applications of WES technology in etiologic diagnosis of ultrasound anomalies in prenatal examination.

## Introduction

The acetylcholine receptor (AChR) is a member of the superfamily of transmitter-gated ion channels and plays a critical role in controlling electrical signals between nerves and skeletal muscle cells. In the embryonic development, AChR consists of one β (*CHRNB*), one δ (*CHRND*), one γ (*CHRNG*), and two α (*CHRNA1*) subunits, but after a gestational age of 33 weeks, the γ subunit is replaced by an ε (*CHRNE*) subunit (Hesselmans et al., 1993). The α subunit of the muscle acetylcholine receptor encoded by *CHRNA1* gene is known as the main target of pathogenic autoantibodies in autoimmune myasthenia gravis.

*CHRNA1* (MIM 100690), *CHRND* (MIM 100720), *CHRNG* (MIM 100730), *RAPSN* (MIM 601592), *DOK7* (MIM 610285), *CNTN1* (MIM 600016), and *SYNE1* (MIM 608441) gene mutations would lead to fetal akinesia deformation sequence and/or multiple pterygium syndrome (MPS) (Chen, 2012), a rare autosomal recessive inherited disorder mainly manifested as arthrogryposis multiplex congenita, pterygia of the neck, fingers, and antecubital, popliteal, and intercrural areas, developmental delay, and facial, vertebral, and genital anomalies (Penchaszadeh and Salszberg, 1981; Ramer et al., 1988). The prevalence of MPS remains uncertain and is supposed to be less than 1/100,000, as reported by a previous study (Mohtisham et al., 2019). MPS is typically divided into prenatally lethal and nonlethal types (Barros et al., 2012; Chen, 2012). The nonlethal form of MPS is also known as Escobar syndrome. The lethal multiple pterygium syndrome (LMPS) is a rare autosomal recessive inherited disorder characterized by intrauterine growth retardation, multiple pterygia, and flexion contractures, causing severe arthrogryposis and fetal akinesia (Vogt et al., 2008; Joshi et al., 2016; Mohtisham et al., 2019). In addition, although the most common inheritance model is autosomal recessive, the autosomal dominant and X-linked inheritance has also been reported (Tolmie et al., 1987; Meyer-Cohen et al., 1999; Chong et al., 2015). Usually, fetuses with LMPS would result in spontaneous miscarriage or stillbirth (Nazari et al., 2019).

In this study, we used whole-exome sequencing (WES) to make a diagnosis of genetic etiology diagnosis in a Chinese family with increased nuchal translucency, fetal edema, fetal neck cystoma, reduced movement, and joint contractures and identified two novel compound heterozygous variants in the *CHRNA1* gene in the fetus, which would lead to LMPS. This study may broaden the spectrum of *CHRNA1* gene variants that lead to LMPS and provide valuable data for application of prenatal WES technology and genetic consultation.

## Case presentation

### Clinical examination

**FIGURE 1:**
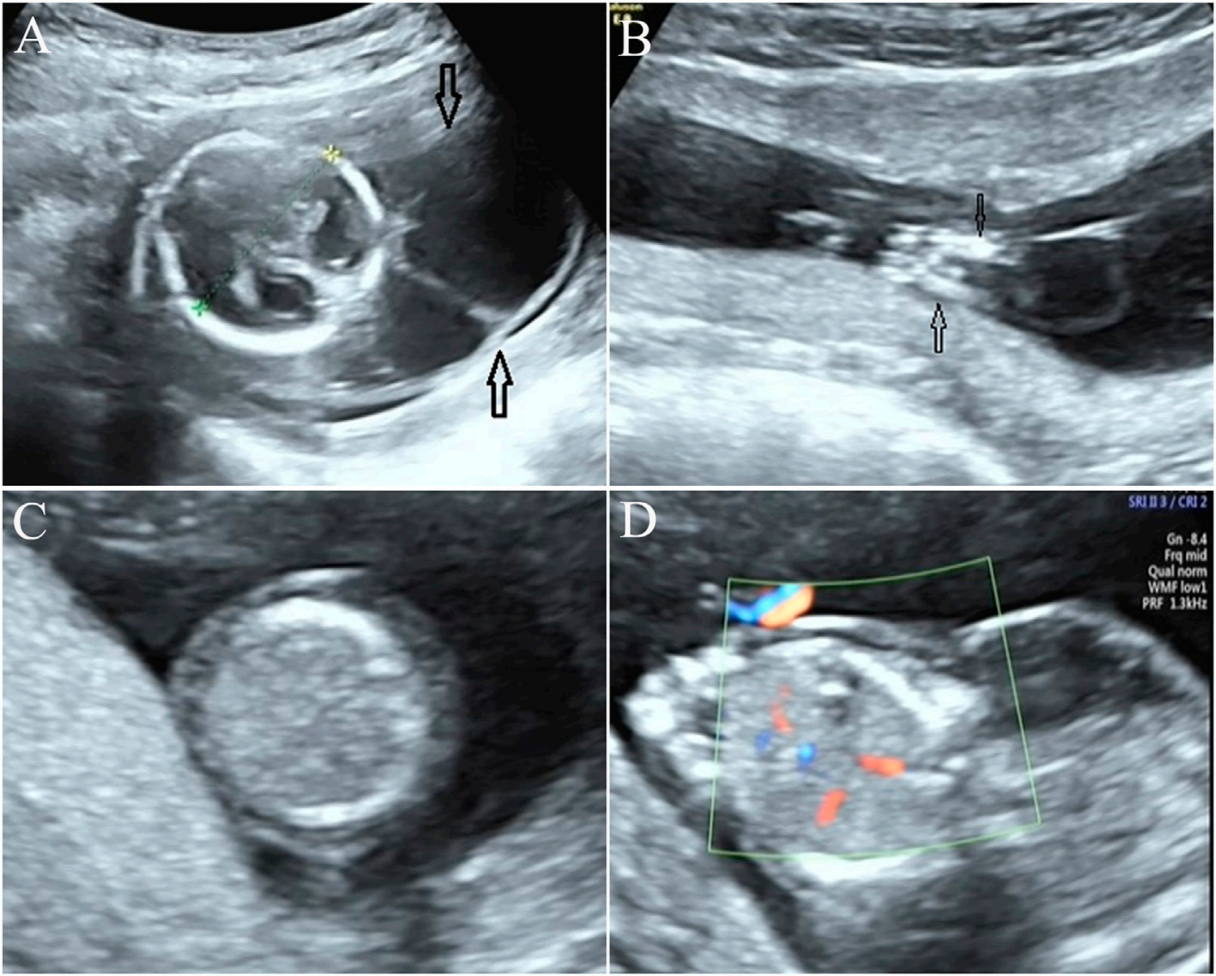
Prenatal ultrasonic examination results of the fetus. **(A)** Ultrasound examination elicited neck water sac tumor in the fetus. **(B)** Approach, flexion, and fixation of both lower limbs were observed in the fetus, indicating joint contractures. **(C,D)**: the trunk skin layer is obviously thickened, and the echo is reduced, and diagnosed as fetal edema.

In this study, a Chinese family with a history of three adverse pregnancies was recruited. The couple denied consanguineous marriage and any related inherited history. This study was approved by the ethics committee of Quanzhou Women’s and Children’s Hospital (2020 No. 31). The three fetuses in this family all had similar ultrasonic abnormalities. Among them, ultrasound of the first pregnancy in the first trimester showed that the fetus had fetal systemic edema, and the pregnant woman and her family chose to terminate the pregnancy without further genetic etiology testing. Subsequently, the woman had the second pregnancy, and ultrasound examination results elicited that the fetus had edema, increased nuchal translucency, neck water sac tumor, reduced movement, and abnormal posture (joint contractures) (Figure 1), and stillbirth occurred at the gestational age of 18^+5^ weeks in the second pregnancy. Upon informed consent from the family, we collected the fetal specimen of the second pregnancy for further cytogenetic and molecular genetic analyses. The third pregnancy of the woman also showed a similar ultrasonic phenotype. Although no stillborn occurred in the second trimester, the family still chose to terminate the pregnancy.

### Molecular analysis

No obvious chromosomal abnormalities and copy number variants were detected by karyotype analysis and chromosomal microarray analysis in the fetus of the second pregnancy, as well as their parents who exhibit normal clinical phenotypes.

**FIGURE 2:**
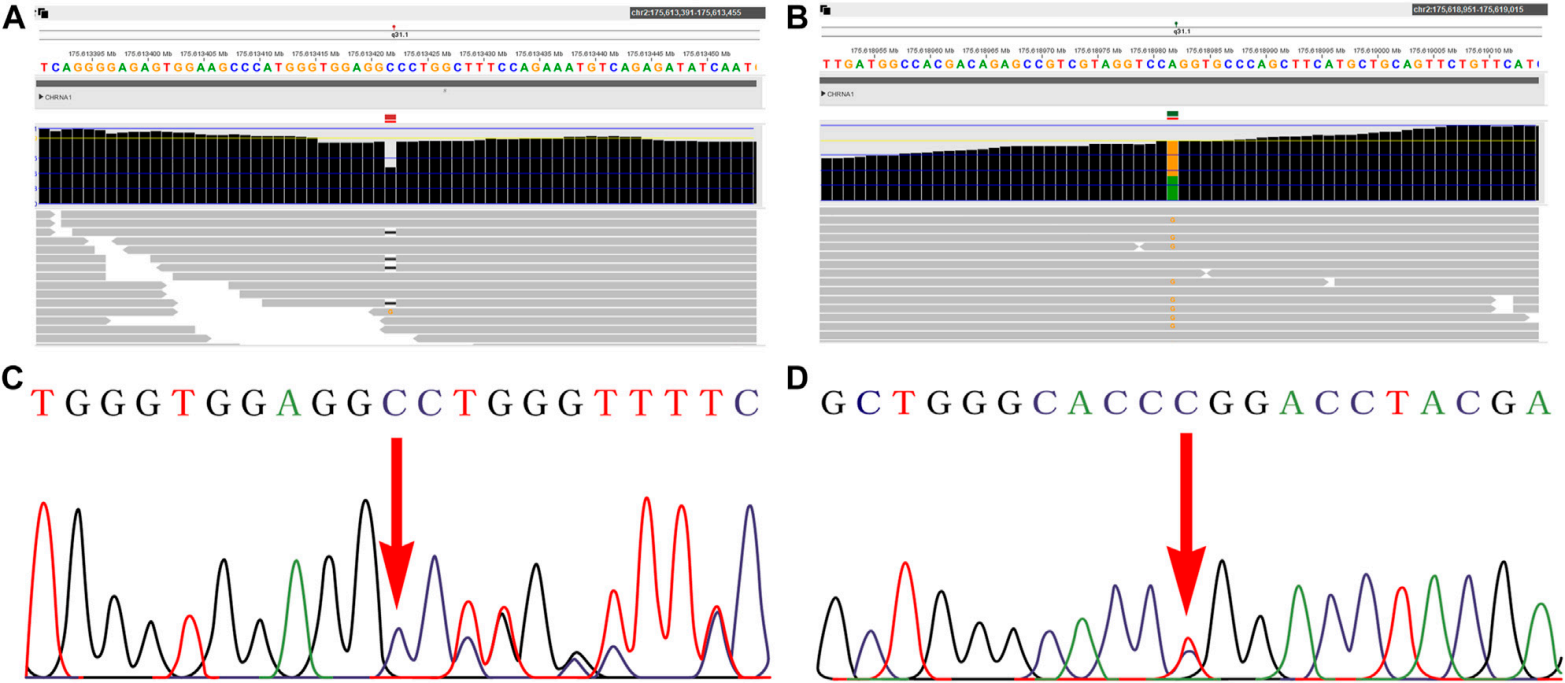
Novel variants of the *CHRNA1* gene identified in the fetus. **A–B**: WES detection results demonstrated novel compound heterozyous variants in the *CHRNA1* gene NM_000079.4: c.[1128delG (p.Pro377Leu*fs*Ter10)]; [505T>C (p.Trp169Arg)] in the fetus. **C–D**: both variants were further confirmed by Sanger sequencing.

WES technology was performed for further genetic etiology in the recruited fetus. A novel frameshift variant in exon 8 compound with a novel missense variant in exon 5 in the *CHRNA1* gene NM_000079.4: c.[1128delG (p.Pro377Leu*fs*Ter10)]; [505T>C (p.Trp169Arg)] was detected in the recruited fetus by WES technology detection, which was inherited from their parents, respectively (Figure 2 and Supplementary Table 1). In the third pregnancy, the compound heterozygous variants were also detected by other hospitals using WES technology. At present, the c.1128delG (p.Pro377Leu*fs*Ter10) variant was absent in the gnomAD (http://gnomad-sg.org/, accession date: 28 June 2022), dbSNP (https://www.ncbi.nlm.nih.gov/snp/?term=, accession date: 28 June 2022), 1000 genomes project (http://browser.1000genomes.org/, accession date: 28 June 2022), PubMed (https://pubmed.ncbi.nlm.nih.gov, accession date: 28 June 2022), ClinVar (https://www.ncbi.nlm.nih.gov/clinvar/, accession date: 28 June 2022) (Table 1), and HGMD (http://www.hgmd.cf.ac.uk/ac/index.php, accession date: 28 June 2022) databases and was not found in the local database as well, but it was interpreted as a likely pathogenic variant (PVS1, PM2_Supporting) according to the ACMG (The American College of Medical Genetics and Genomics, ACMG) guidelines (Richards et al., 2015). In addition, the c.505T>C (p.Trp169Arg) variant was also absent in the databases mentioned earlier, and online computer-aided analysis predictions (http://159.226.67.237/sun/varcards/welcome/index) suggest that this variant is more likely to affect the protein structure/function (damaging score: 0.87). According to the ACMG guidelines (Richards et al., 2015), the c.505T>C (p.Trp169Arg) variant was interpreted as variant of uncertain significance (PM3, PM2_Supporting, and PP3).

**TABLE 1:** Variants of the *CHRNA1* gene and related clinical findings in the ClinVar database.^a^

Variant (NM_000079.4)	Protein	ACMG classification	Clinical phenotype	Mutation type
c.518dup	p.Ser174Leu*fs*Ter194	P/LP	LMPS	Frameshift mutation
c.436_437insTG	p.Ser146Met*fs*Ter20	VUS	NP	Frameshift mutation
c.380_381del	p.Lys127Ser*fs*Ter18	LP	Congenital myasthenic syndrome	Frameshift mutation
c.292dup	p.Ile98Asn*fs*Ter17	P	LMPS	Frameshift mutation
c.779-1_779insA	—	LP	LMPS	Splicing mutation
c.779-2A>C	—	LP	NP	Splicing mutation
c.235-1G>A	—	LP	NP	Splicing mutation
c.1345C>T	p.Arg449Ter	VUS	NP	Nonsense mutation
c.844G>T	p.Glu282Ter	P	LMPS	Nonsense mutation
c.370A>T	p.Lys124Ter	P	LMPS	Nonsense mutation
c.317G>A	p.Trp106Ter	P/LP	NP	Nonsense mutation
c.249C>A	p.Tyr83Ter	P	LMPS	Nonsense mutation
c.175C>T	p.Gln59Ter	P	NP	Nonsense mutation
c.166C>T	p.Gln56Ter	LP	NP	Nonsense mutation
c.1128delG (our study)	p.Pro377Leu*fs*Ter10	LP	LMPS	Frameshift mutation
c.505T>C (our study)	p.Trp169Arg	VUS	LMPS	Missense mutation

a As shown in Table 1, the overall frameshift, splicing, and nonsense mutations in the *CHRNA1* gene in the ClinVar database were presented, except for the 153 missense mutations. P: pathogenic; LP: likely pathogenic; VUS: variants of uncertain significance; NP: not provided; LMPS: lethal multiple pterygium syndrome.

## Discussion and conclusion

In the clinical practice, chromosomal microarray analysis (CMA) or copy number variation sequencing has been increasingly used to assess the genetic cause in miscarriage and stillbirth (Sahlin et al., 2014; Martinez-Portilla et al., 2019; Marquès et al., 2020; Zhang et al., 2021). A recent meta-analysis of seven studies involving 903 stillborn fetuses demonstrated a 4% incremental yield of pathogenic copy number variants of CMA over karyotyping, among which 22q11.21 deletion was the most common variant responsible for stillbirth (Martinez-Portilla et al., 2019). However, more than half of them could not get a clear genetic diagnosis. WES has obvious advantages in the detection of monogenic diseases and is, therefore, suggested to be used in prenatal genetic etiology diagnosis in fetuses with ultrasonic structural abnormalities (Lord et al., 2019; Petrovski et al., 2019). In the present study, we detected two novel *CHRNA1* gene mutations in the stillbirth fetus that may result in LMPS by WES.

Previous studies have indicated the application value of WES in identifying the genetic etiology for pregnancy loss or stillbirth (Fu et al., 2018; Demetriou et al., 2019; Zhao et al., 2021). More studies have identified *CHRNA1* mutations in fetuses with recurrent pregnancy loss or stillbirth using WES. A systematic review of 50 studies (Colley et al., 2019) reported a range of candidate genes (*CHRNA1*, *DYNC2H1*, and *RYR1*) that may induce pregnancy loss. In addition, a recent study reviewed 15 articles of 74 families including 279 reported recurrent pregnancy loss, identified 34 candidate pathogenic variants in 19 genes including *CHRNA1* gene by exome sequencing, and recommended that trio-based exome sequencing can be performed in cases with recurrent pregnancy loss and with normal parental karyotypes (Robbins et al., 2019). In addition, a novel mutation in *CHRNA1* was identified by exome sequencing, which was suggested as the cause for recurrent fetal loss, and they hypothesized that exome sequencing could disclose the underlying autosomal recessive mutations in families with recurrent fetal loss (Shamseldin et al., 2013). In the present case study, we identified two compound heterozyous variants in the *CHRNA1* gene, which further confirms the application value of WES in genetic analysis of recurrent pregnancy loss or stillbirth.

The nicotinic acetylcholine receptor (AChR) has five subunits of four different types: two alpha subunits and one each of beta, gamma (or epsilon), and delta subunits, which control electrical signaling between nerve and muscle cells by opening and closing a gate (Miyazawa et al., 2003). Among them, *CHRNA1* encodes two alpha subunits, playing an important role in maintaining the AChR structure. As exhibited in the OMIM database, heterozygous mutations of *CHRNA1* can cause autosomal dominant congenital slow-channel myasthenic syndrome and congenital fast-channel myasthenic syndrome, while homozygous mutations or compound heterozygous mutations of *CHRNA1* would lead to autosomal recessive LMPS. A previous study identified homozygous mutations in the *CHRNA1* gene in patients from two families with LMPS (Michalk et al., 2008). Among them, the homozygous c.761G>T (p.Arg234Leu) mutation was identified in both fetuses in family 1 with similar prenatal ultrasonic features including growth delay, edema, cystic hygroma, decreased movements, and joint contractures. They observed that the first fetus of family 1 was a stillbirth at a gestational age of 24 weeks, and the second fetus was terminated at a gestational age of 20 weeks. Like family 1, similar intrauterine problems were displayed in both fetuses of family 2, who carried the homozygous mutation of c.117-133dup17 (p.His25Arg*fs*X19) in the *CHRNA1* gene. In addition, a study conducted by Dickinson et al. (2016) found that homozygous lethal mutation was observed in the *CHRNA1*-knockout mice. In the present study, compound heterozygous variants of NM_000079.4: c.[1128delG (p.Pro377Leu*fs*Ter10)]; [505T>C (p.Trp169Arg)] in the fetuses were detected, who manifested intrauterine edema, increased nuchal translucency, neck water sac tumor, and joint contractures, which are consistent with the intrauterine clinical features of LMPS.

At present, neither of two novel *CHRNA1* variants identified in this study has been reported in the databases or the literature. Among them, the missense variant was classified as the variant of uncertain significance according to the ACMG guidelines. Given the consistence of the clinical phenotypes in the fetuses with LMPS and the genotype–phenotype segregation present in the family, we believe that both variants detected in *CHRNA1* could be pathogenic variants and may lead to LMPS. However, future functional studies are required to clarify the molecular mechanism.

In conclusion, an etiologic diagnosis was conducted successfully in a Chinese family with recurrent fetal edema, fetal neck cystoma, and joint contractures by WES. This is the first study reporting the identification of two novel compound heterozyous variants in the *CHRNA1* gene that may lead to LMPS. Our findings may broaden the spectrum of *CHRNA1* gene mutations that result in LMPS and provide valuable data for the application of the prenatal WES technology and genetic consultation.

## Data Availability

The datasets for this article are not publicly available due to concerns regarding participant/patient anonymity. Requests to access the datasets should be directed to the corresponding authors.
