# Supplementary material for: Case Report: Novel compound heterozygous variants in CHRNA1 gene leading to lethal multiple pterygium syndrome: A case report
Source: Front Genet. 2022 Aug 26;13:964098. doi: 10.3389/fgene.2022.964098 (PMC9459375; doi:10.3389/fgene.2022.964098)
Supplement: Supplementary file 1 [file Table1.DOC]

| Variants | | |  | Primers 5’→3’ | |
| --- | --- | --- | --- | --- | --- |
| CHRNA1NM_000079:c.505T>CFGCTGTCTCCTGTTAACGTCCRAGCTCCCATTTAGTTCTCCCTTCHRNA1NM_000079:c.1128delGFTTATGCTTGCCTCCTGGACARCTAAGGTGGTCTAGAGGCGG | | |  |  | |
|  | |  |
|  |  |  | | | |
|  |  | | | |
